# Interference between PARPs and SIRT1: a novel approach to healthy ageing?

**DOI:** 10.18632/aging.100326

**Published:** 2011-05-07

**Authors:** Carles CantÓ, Johan Auwerx

**Affiliations:** Ecole Polytechnique Fédérale de Lausanne; CH-1015 Lausanne, Switzerland

**Keywords:** aging, metabolism, longevity, sirtuins, PARP

## Abstract

Poly(ADP-ribosyl) polymerases (PARPs) have traditionally been linked to chromosome maintenance and DNA repair. Recent findings identify PARPs as key modulators of metabolism through their influence on SIRT1 activity, hinting to a possible role of PARPs as longevity regulators.

Defects in energy metabolism, and, more specifically, mitochondrial function are intimately linked to the general decline in physiological functions during ageing [1]. While generally thought to be a consequence of random damage, the hypothesis that ageing should also be considered a metabolic state is gaining importance, as defective energy metabolism and DNA damage seem intimately intertwined. Indeed, a flurry of recent reports illustrates how DNA damage influences metabolic homeostasis by affecting mitochondrial function. A nice example of such a relationship was provided by the demonstration that dysfunction of telomere maintenance could impact on mitochondrial function [2]. The DNA damage induced by deficient telomere capping would decrease the activity of PGC-1a, a master transcriptional regulator of mitochondrial biogenesis, via a pathway that involved p53 activation.

Recent observations from our lab [3, 4] can complement the observations from Sahin et al and introduce a matching mechanism by which DNA-damage can influence mitochondrial biogenesis. This new pathway involves the interplay between two enzymes, i.e. the Poly(ADP-ribose) polymerases (PARPs) and the sirtuins. Both enzyme families are strongly conserved in eukaryotes and share the particularity of using NAD^+^ as a cosubstrate, which is converted into nicotinamide (NAM) by the respective enzymatic activities [5]. PARP activity is mainly attributed to PARP-1, a ubiquitous and abundant nuclear protein. PARP-1 can bind to various DNA structures and nucleosomes, and has important roles in DNA repair, chromosome stability and transcriptional regulation (for review, see [6]). The reaction catalyzed by PARP-1 uses NAD^+^ to form polymers of ADP-ribose units onto acceptor proteins, cleaving off NAM. Cellular poly(ADP-ribose) (PAR) formation is dramatically stimulated upon DNA damage, and, as a result, NAD^+^ levels plummet when DNA is damaged [6, 7]. While PARP-1 accounts for 90% of PARP activity under genotoxic situations, a second enzyme, PARP-2 is responsible for the remaining 10% [8, 9]. The role and activation of other PARP family members under such circumstances remains unexplored.

SIRT1, the first mammalian member of the sirtuin family identified, is a NAD+-depedent deacetylase that regulates diverse aspects of global metabolism by regulating the acetylation status and activity of a number of metabolic enzymes and transcriptional regulators [10]. Notable amongst these substrates is PGC-1a. SIRT1-mediated deacetylation of PGC-1a enhances its capacity to coactivate transcription of nuclear encoded mitochondrial genes [11]. SIRT1 activity can be controlled by the regulation of its expression, by some post-translational modifications and, interestingly, by NAD^+^ levels [5]. The K_m_ of SIRT1 for NAD^+^ is around the physiological concentration range of NAD^+^, implying that in diverse circumstances, NAD^+^ can rate-limit SIRT1 activity [5].

Due to this particular characteristic, SIRT1 has been proposed to act as an intracellular NAD^+^ sensor that translates changes of the metabolic/redox state of the cell into adaptive transcriptional responses [12]. Furthermore of relevance is that the breakdown product of both the SIRT1 and PARP reactions, NAM, is an allosteric inhibitor of SIRT1 activity [13].

The use of NAD^+^ as a common cosubstrate has in the past led to propose a possible interrelationship between PARP and SIRT1 activities [14]. In two recent reports, we have established how this interrelationship has strong metabolic implications [3, 4]. The deletion of either the PARP-1 or PARP-2 gene in mice enhances SIRT1 activity, which leads to a higher mitochondrial content, ability to oxidize fatty acids, ultimately culminating in the prevention against diet-induced obesity. Interestingly, PARP-1 and PARP-2 do so via very different mechanisms. The deletion of the PARP-1 gene or the inhibition of PARP-1 activity boosts cellular NAD^+^ content [3]. This effect on NAD^+^ is not immediate, probably because PARP-1 activity is very low in cells/tissues in basal conditions, and it requires a protracted inhibition of PARP-1 to promote significant effects on NAD^+^ accumulation. This enhanced NAD^+^ content then activates SIRT1, leading to the deacetylation of key targets that induce mitochondrial biogenesis [3]. In contrast to NAD+ levels, NAM levels were not affected by PARP-1 deletion [3]. Conversely, when cells are subjected to oxidative stress by exposure to H_2_O_2_, PARP-1 is activated and SIRT1 activity is robustly reduced, as PARP-1 activation limits NAD^+^ bioavailability [3, 7, 15]. Treatment with PARP inhibitors in these circumstances allows the cell to maintain NAD^+^ levels and SIRT1 activity [3]. Collectively with the fact that SIRT1 is not a substrate for and regulated by PARylation [3], these observations indicate that PARP-1 is a gatekeeper for SIRT1 activity by limiting NAD^+^ availability.

The case of PARP-2, however, is different. In line with its relatively minor role in global PARP activity [8, 9], the reduction of PARP-2 levels does not influence NAD^+^ levels in the cellular models [4]. Surprisingly, and similar to PARP-1, a reduction of PARP-2 levels were nevertheless also linked to SIRT1 activation [4]. This increase in SIRT1 activity, rather than being the consequence of a modulation of NAD^+^ levels, was attributed to a higher expression level of SIRT1 [4]. In fact, PARP-2 binds directly to the SIRT1 promoter and represses its transcription [4]. Via this way, PARP-2 gene deletion induced SIRT1 levels in all tissues and cell models tested, subsequently promoting mitochondrial biogenesis, enhancing the ability to oxidize fat and preventing against the onset of diet-induced obesity [4]. Therefore PARP activation influences SIRT1 activity through two different means, i.e by limiting NAD^+^ availability (PARP-1) and by regulating SIRT1 expression at the transcriptional level (PARP-2) (Figure1).

**Figure 1.**
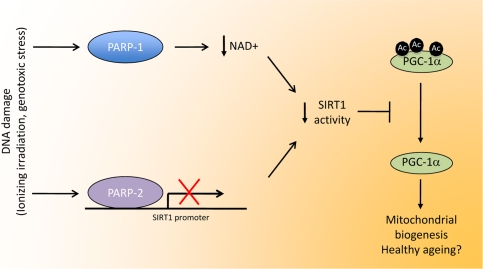
Linking DNA damage to mitochondrial biogenesis. Upon DNA damage, PARP activity in the cell is highly enhanced. The activation PARP-1, the enzyme responsible for most PARP activity, would lead to NAD^+^ depletion, therefore limiting SIRT1 activity by lowering the bioavailability of this crucial coenzyme. PARP-2 binds to the SIRT1 promoter, inhibiting transcription, resulting in reduced SIRT1 levels. In all, both enzymes would co-ordinately lead to a decrease in SIRT1 activity through different means. The reduction in SIRT1 activity would reduce the deacetylation rate of several transcriptional regulators, such as PGC-1a. The resulting hyperacetylation of PGC-1a would then decrease mitochondrial-related gene expression, having a strong impact on metabolic disease and, potentially, a healthy ageing.

The above results suggest a novel mechanism by which DNA damage influences mitochondrial function and global metabolism. The binding of PARP-1 to DNA strand breaks and its subsequent catalytic activation might limit SIRT1 activity and, therefore, impede the correct maintenance of mitochondrial homeostasis. This relationship between PARPs and SIRT1 might not only affect metabolic control but also impact on aging. In fact, in lower eukaryotes, SIRT1 activity influences longevity and has been proposed to be a mediator of the effects of calorie restriction on lifespan extension [16]. Future research will have to establish whether PARP deletion/inhibition might alter lifespan by enhancing SIRT1 activity. One common criticism on this hypothesis is that reduced PARP activity might compromise DNA repair and maintenance. Therefore, a protracted decrease in PARP activity might be detrimental for proper cellular and organismal survival. While this is a fair hypothesis, it is also true that PARP-1 or PARP-2 deficient mice do not show signs of enhanced DNA damage [17, 18]. Such a phenotype only becomes evident when these mice are exposed to ionizing irradiation or chemically-induced genotoxic stress [18, 19]. Interestingly, the simultaneous knock.out of both PARP-1 and PARP-2 results in embryonic lethality, indicating that PARP-1 and PARP-2 can compensate to a certain extent the key functions of each other in basal genomic maintenance and developmental processes [19].

A role for PARPs on longevity had been already postulated almost two decades ago, when experiments in permeabilized mononuclear leukocytes of 13 different species revealed a positive correlation between maximal PARylation capacity and longevity [20]. PARylation capacity was not the consequence of higher PARP-1 enzyme content, but attributed to intrinsic differences in PARP activity [20]. Additionally, it was observed that PARP activity declined in aging mononuclear leukocytes [20, 21]. These premises led to the speculation that enhanced PARP activity would allow longevity [22], which is diametrically opposite to the speculation derived from our studies [3, 4]. How might this discrepancy be explained? First of all, it must be noted that the studies on PARP activity and longevity do not enable to establish a cause-effect relationship. Indeed, recent gain of function studies in mice which overexpress an additional copy of the human PARP-1 gene (hPARP-1 mice), demonstrated that increased PARP-1 gene dosage actually led to a premature occurrence of age-related diseases [23]. Many inflammation-related pathologies, such as glomerulopathy, nephritis, hepatitis, myocarditis/cardiac fibrosis, pneumonitis, and dermatitis, that naturally occur in old wild type mice develop in hPARP-1 mice at younger ages and with higher frequency and severity, leading to a reduced median lifespan [23]. This suggests that the higher PARP-1 activity in leukocytes from long lived individuals is rather a consequence but not the cause of longevity. Furthermore, it is difficult to evaluate whether the higher PARP-1 activity observed in the original experiments in leukocytes can be extrapolated to other cell types and tissues, including slowly dividing differentiated tissues.

Our results obtained in the PARP-1 and PARP-2 knockout mice would also predict that higher PARP-1 gene dosage would compromise metabolic fitness. In line with the predictions from our studies, mice overexpressing hPARP-1 were heavier than their wildtype littermates [23], an effect which was at least in part caused by increased fat mass and which was accompanied by glucose intolerance. These results consolidate our findings indicating that low PARP activity enhances metabolic fitness, prevents inflammation-related diseases and, potentially, extends health/lifespan. It will be, however, necessary to perform health/lifespan studies in organisms with chronically reduced PARP activity in order to rigorously test this hypothesis. This will not be an easy task, as some eukaryotes only have one PARP homolog and its deletion is lethal [24]. Similarly, in other organisms PARP homologs are dispensable for life, but their role in genomic maintenance might be different from that of the mammalian PARPs [25]. In addition, it will be interesting to understand to which extent sirtuins and/or NAD^+^ homeostasis will be key to explain the possible relationship between PARPs and longevity. In this latter regard, it is notable that PARP deletion on mice has different, even opposite effects, depending on the mouse strain used [3, 18, 26, 27]. This might underscore strain-dependent differences in NAD^+^ metabolism, which requires future investigation. Indeed, C57Bl/6J, the most widely used mouse strain is defective for nicotinamide nucleotide transhydrogenase (Nnt) [28, 29], prompting a higher susceptibility to develop defects in insulin secretion and altered glucose homeostasis. Nnt is a inner mitochondrial membrane protein that catalyzes the interconversion of NADH and NADPH in the mitochondrial matrix. The consequences that Nnt deficiency might really have on NAD^+^ homeostasis are, however, not clear [5]. Furthermore, the fact that reducing PARP-1 activity with different genetic and pharmacological strategies in diverse cellular models faithfully recapitulates the effects observed in vivo clearly argues that the phenotype in the C57Bl/6J is genuine and not consequent to Nnt deficiency, and consolidates previous observations indicating that the Sv129 mouse strain is not optimal to evaluate metabolic disease phenotypes [30, 31]. Finally, it will also be important to identify the scenarios in which PARP activity might be balancing metabolism and genomic maintenance.

Despite these novel and tantalizing findings, the many remaining open questions clearly illustrate that, half a century after their initial discovery [32], PARPs still have many aces up their sleeves. The quest to uncover the integral picture of PARP biology will warrant future research for yet some more time.
